# Governance using the water-food-energy nexus and human-factor measures

**DOI:** 10.1371/journal.pone.0261995

**Published:** 2022-01-27

**Authors:** Shaul Sorek, Aviva Peeters, Fany Yuval, Dragan Savic

**Affiliations:** 1 Zuckerberg Institute for Water Research, J. Blaustein Institutes for Desert Research, Ben-Gurion University of the Negev, Midreshet Ben-Gurion, Israel; 2 J. Blaustein Institutes for Desert Research, Ben-Gurion University of the Negev, Midreshet Ben-Gurion, Israel; 3 Department of Public Policy & Administration, Faculty of Business & Management, Ben-Gurion University of The Negev, Beer-Sheva, Israel; 4 KWR Watercycle Research Institute, Nieuwegein, The Netherlands; 5 University of Exeter, Exeter, United Kingdom; Shenzhen University, CHINA

## Abstract

Household water food and energy (WFE) expenditures, reflect respective survival needs for which their resources and social welfare are inter-related. We developed a policy driven quantitative decision-making strategy (DMS) to address the domain geospatial entities’ (nodes or administrative districts) of the WFE nexus, assumed to be information linked across the domain nodal-network. As investment in one of the inter-dependent nexus components may cause unexpected shock to the others, we refer to the WFE normalized expenditures product (Volume) as representing the nexus holistic measure. Volume rate conforms to Boltzman entropy suggesting directed information from high to low Volume nodes. Our hypothesis of causality-driven directional information is exemplified by a sharp price increase in wheat and rice, for U.S. and Thailand respectively, that manifests its impact on the temporal trend of Israel’s administrative districts of the WFE expenditures. Welfare mass (WM) represents the node’s Volume combined with its income and population density. Formulation is suggested for the nodal-network WM temporal balance where each node is scaled by a human-factor (HF) for subjective attitude and a superimposed nodal source/sink term manifesting policy decision. Our management tool is based on two sequential governance processes: one starting with historical data mapping the mean temporal nodal Volumes to single out extremes, and the second is followed by WM balance simulation predicting nodal-network outcome of policy driven targeting. In view of the proof of concept by model simulations in in our previous research, here HF extends the model and attention is devoted to emphasize how the current developed decision-making approach categorically differs from existing nexus related methods. The first governance process is exemplified demonstrating illustrations for Israel’s districts. Findings show higher expenditures for water and lower for energy, and maps pointing to extremes in districts’ mean temporal Volume. Illustrations of domain surfaces for that period enable assessment of relative inclination trends of the normalized Water, Food and Energy directions continuum assembled from time stations, and evolution trends for each of the WFE components.

## Introduction

The Nexus concept defines mutual interactions between group of components and feedback mechanisms that cannot be resolved by studying each component independently. Any interventions to improve performance of one of the components may affect the performance of the others. Furthermore, the inter-dependence is complex, and difficult to predict using non-quantitative methods [1].

A study conducted for NASA [2] showed that: “…*global industrial civilizations could collapse in coming decades due to unsustainable resources exploitation and increasingly unequal wealth distribution*". In addition, "*the project identifies the most salient interrelated factors which explain civilizational decline*, *and which may help determine the risk of collapse today*: *namely*, *Population*, *Climate*, *Water*, *Agriculture*, *and* *Energy*".

Integration of management and governance among different sectors and scales are advocated to maintain the security and sustainability of WFE (synonymous with WEF) resources [3–5]. Although the WFE Nexus represents some of the most vital aspects of mankind’s survival, decisions on how to quantitatively manage WFE resources are often made based on subjective or qualitative understanding of the situation.

The security of WFE resources is becoming a matter of concern [6]. However, although our future depends on the intricate interplay of all three WFE resources, many of the reported studies [7–14] have focused only on the individual resources of this Nexus (e.g., national water resources, virtual water trade or food trade). The intricate interaction among the WFE components and the system’s overall behavior on a national scale are poorly understood and a comprehensive Nexus-modeling platform is missing [15–17]. This knowledge gap is even more relevant when future change and shocks to any single water, food or energy component of the WFE Nexus is considered. Such complex interaction of the WFE components can only be thoroughly understood through a representative quantitative holistic approach. The knowledge gained through such an approach can enhance decision-making processes for efficient and sustainable system management–a challenge which must be advocated at the international level [6].

Current developments in nexus planning addressing the modeling between inputs and outputs associated with socio-ecological system, are presented in Nhamo et al [18, 19].

A computer-based decision support system (DSS) for comprehensive management of the WFE Nexus can assist in solving complex spatiotemporal-related problems of multiple criteria and in evaluating different quantitative compared scenarios for informed policy driven decision-making. One of the major challenges of such a system, however, is the need for big data mining and big data analytics through which a management tool can be developed for integrated water resource management and policy (IWRMP). In IWRMP we refer to a policy that also addresses preferences between central versus peripheral nodes and urban versus rural zones, and focus on modeling one attribute being Water (e.g. predicting temporal economic efficiency via expenditure -vs- allocation and sustainability via water quantity/quality), integrated with quantitative scaling factors (e.g., climatic conditions, subjective attitude, soil quality, percentage of water-impervious surfaces and population density, i.e., the ratio of a node’s population number per its area). For decision making based on Nexus for instance with water as one of the Nexus components, the focus is on interdependent principal attributes, while also considering the IWRMP factors. Resource administrators should coordinate criteria associated with different stakeholders against operational alternatives utilizing mathematical procedures, taking into account the characteristic conditions at chosen domain nodes [20].

Decision-making on complex and interconnected hydrological, environmental, and socio-economic variables [21–25] implements DSS for IWRMP to develop, evaluate, plan and monitor policy decisions [26]. Scaling the relative importance of the WFE components by accounting for the human-factor (HF) is also essential for decision-making. However, we are not aware of any studies integrating subjective and objective perspectives of WFE in a quantitative manner under the same framework.

We suggest employing decisions based on quantifiable measures rather than on decisions based solely on objective considerations [27–29].

The basis of our strategy is to develop decision-making cycles addressing the WFE Volume as one composed quantity by using visualizations, followed by simulations with ordinary differential equation (ODE) subject to information divergence between nodes, which sets this research apart form previous work concerning this subject. This approach is different than other reported quantitative WFE-related studies such as: assessments in Xiaodong and Vesselinov [30] to ascertain the relationships between the components, and apply a linear programming model for managing the WFE Nexus in a way that minimizes global financial cost of the system; [31] who uses linear algebraic equations due to separate inputs of the WFE resources balancing direct and indirect effects of planning scenarios; the system dynamics modeling (SDM) applied by Hussain et al. [32] for related consumption at the household level; survey-based qualitative policy strategies [33]; framework/methodology for creating integrated production (food, energy, usable water, etc.) systems on a local scale [34]; a hydro-economic model examining the tradeoff in water/energy supply from two potential operation modes [35]; qualitative analysis of existing regulatory structures [36]; application of a generalized method of moments modeling to obtain reliable parameter estimates creating a food security index [37]; a review of an existing policy conceptual framework to address the Nexus in terms of livelihoods [38], and of the possibility of weighting the components equally instead of emphasizing primarily water [39], and a research overview highlighting challenges and identifying future research opportunities in terms of process systems engineering [40].

Herein, the developed management tool addresses practical implications and allows for predicting the outcome of strategic quantifiable interventions to domain geospatial nodes. The applicability of the approach and its potential is demonstrated on historic data for which the rational and ideas of the essence of the DMS and model are described next.

## Storyline

The objective was to establish a DMS that based on quantifiable assessments, helps managers determine the best policy driven modifications to rectify social welfare imbalances in WFE expenditures across a domain nodal-network.

**Fig 1:**
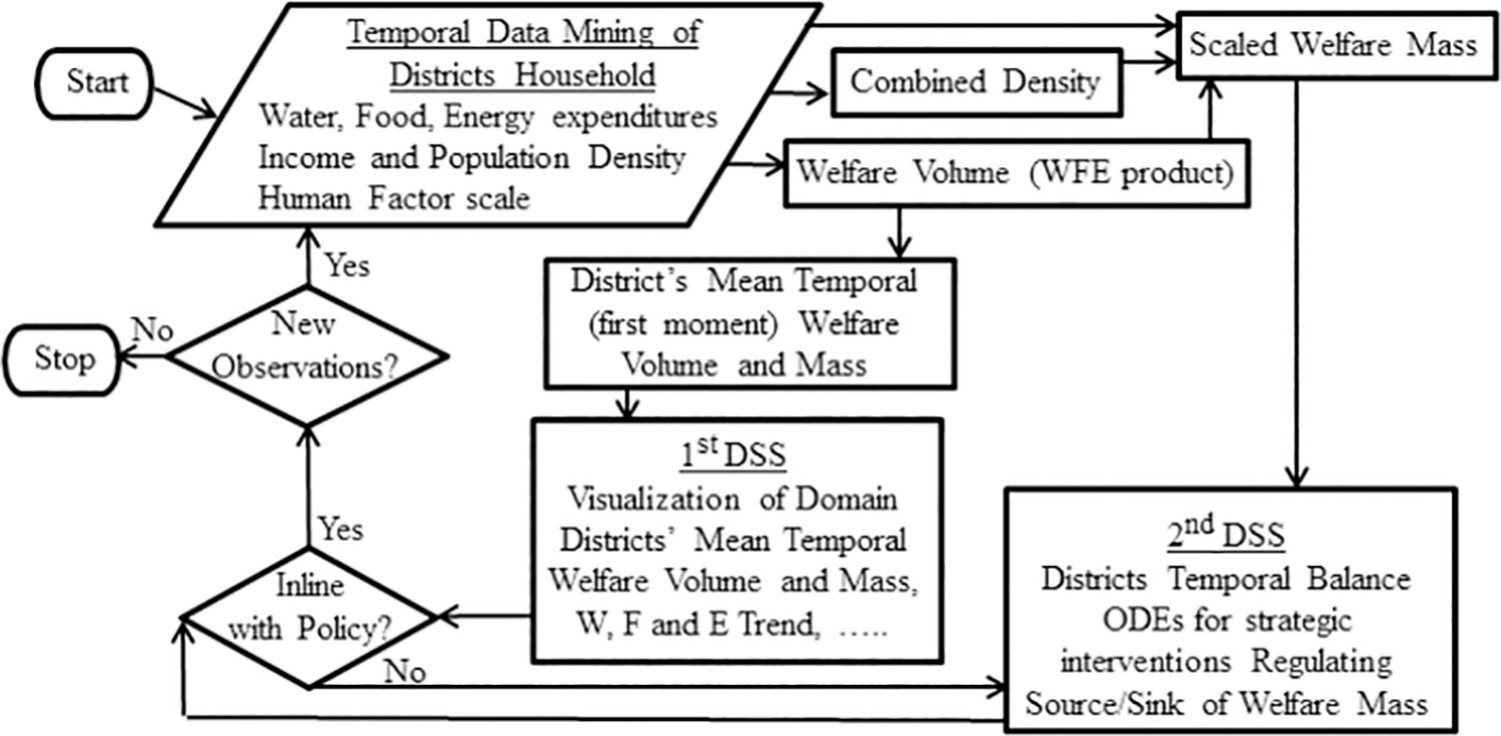
Flowchart of the macro management tool for WFE Nexus of geospatial administrative nodes (districts) with core aspects addressed by two DSS processes. The first for the “Realize” and “Why” phases and the second for the”How” phase.

In the context of this study, management is represented by a domain authority allocating investments based on spatiotemporal resolution with feedback control. Addressing this, Fig 1 describes the developed macro management tool that involves cycles of DMS, where data mining serves as the basis for identifying the underlying problem. The cycle starts (Fig 1) with the 1^st^ DSS process based on historical data mining to evaluate Volume quantities that on the basis of illustrations addresses the “Realize” phase (i.e., identifying undesirable node Volume value), and the “Why” phase (i.e., the relative weight of the normalized components causing this undesirable node Volume value) in order to understand the driving forces.

Once these are identified, the process moves to the 2^nd^ DSS process to address the "How" phase (i.e., quantitative means of rectifying the undesirable node Volume value) in which WM quantities are simulated to regulate possible corrective measures.

Quantitative assessment is a core aspect which this manuscript elaborates on extensively in terms of Nexus management modeling. In conjunction with Fig 1, spatiotemporal management is determined by WFE Volume distribution over regional nodal (geospatial entity) network. Policy driven decision making starts with visual comparisons of these Volumes. Quantitative interventions to rectify future WM imbalances, leading to sustainable optimal regulation, is achieved by solving WM balances subject to imposing source/sink terms over the nodal network. Each proceeding time step yields the nodal predicted WM load from which water allocation is decided, guiding the food and energy allocations [41].

**Fig 2:**
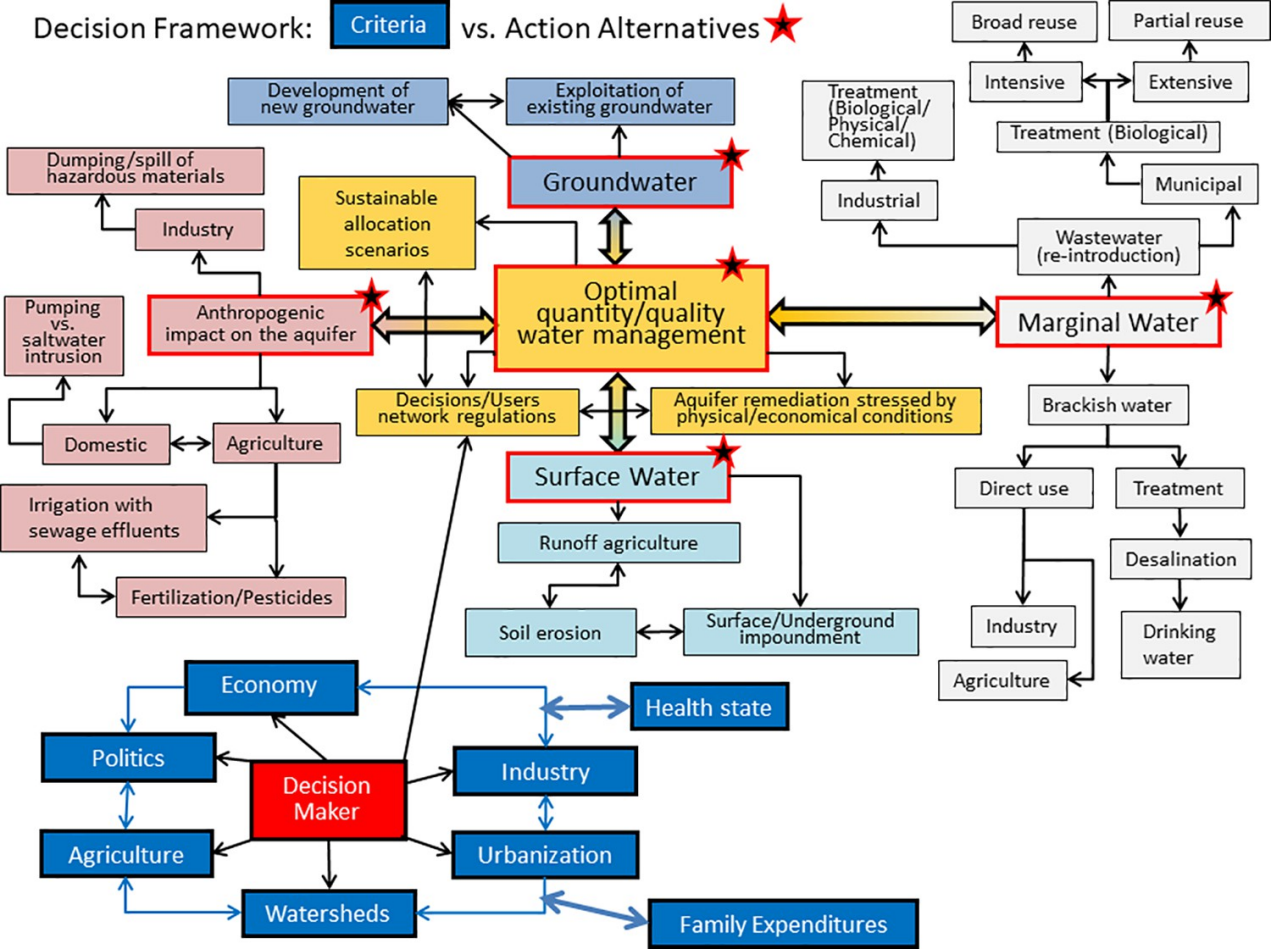
Possible core, water related, action alternatives interlinked with criteria (i.e., decision framework) and associated with the WFE nexus. Alternatives (emphasized by different colors) are associated with sub-units.

Moreover, the complexity of Nexus driven decision making for water-related systems which can account for different criteria and their related state varaibles is emphasized (section 4) and described (Fig 2).

A survey of existing decision making approaches (sections 3) reveals knowledge gaps being:

1) models focus on the influence of the individual WFE components which is contradictory to accounting for their interdependencies being a virtue of the Nexus essence; 2) models adopt an indirect integration between the WFE components across geospatial locations and don’t account in a quantitative way for the subjective attitude in the models’ framework; 3) no illustration of the temporal evolution in the context of the Water, Food and Energy directions continuum at the investigated domain and 4) at such a domain, doubt as to where, when and to what degree is policy driven intervention required. Targeting policy driven interventions per locations are not possible when evaluating performance indicators and indices [42].

Accordingly, our developed management model addresses these drawbacks by:

1) using the human-factor (HF) in the quantitative temporal nodal-network as the subjective attitude (survey of its importance discussed in section 4); 2) introducing the normalized WFE expenditure components product (Volume) as the nexus holistic measure, into the location-based nodal-network; 3) establishing a management tool (Fig 1) that addresses: i) the spatiotemporal mapping of historic WFE values over the investigated time period. This enables to focus on Volume-extreme nodes (Figs 4–6), and domain WFE expenditure surfaces for relative inclination trends on the normalized components continuum with nodes’ temporal track samples (Fig 7A) and their temporal evolution ordered from the northern district to the southern one (Fig 7C); ii) temporal lumped parameter modelling (subsection 4.1) using ODEs to predict WM balance across the domain nodal-network and account for WM divergence and thus enabling to target policy driven source/sink terms that can rectify extreme nodal values.

### DSS for IWRMP

A DSS for IWRMP integrates five core features: (*i*) a data-acquisition system; (*ii*) a user-data-model interface; (*iii*) a database; (*iv*) a data-analysis tool; and (*v*) a set of interconnected models [43].

A number of DSS have been developed for IWRMP. Kelly et al. [44] reviewed five types of common integrated modeling approaches, three of which are: (*i*) a system dynamic modeling (SDM) approach [44–48] that does not require algorithmic codes, interprets how changes in one part of the system impact the system as a whole, and gives guidance for considering alternative scenarios. However, one major limitation of this approach is that it does not support the representation of spatial variables and spatial variability in the modeled system, for which geographical information systems (GIS) is suggested; (*ii*) the Bayesian-networks approach which is suitable for the system’s uncertain inputs and outputs [22, 44, 49–51], and enables the use of objective and subjective data, provides an empirical solution, and can be continuously updated to include new information; (*iii*) an agent-based modeling (ABM) approach which models social interactions between heterogeneous agents in a system, distributed spatially across a shared physical environment [52–61] and accounts for influences exerted by agents in the system as well as the effects of such influences on particular agents. Yet, ABM inhibits the trade-off between representational accuracy and the tractability of its analysis. A major concern in developing and implementing a DSS for IWRMP is that it requires a careful consideration of the DSS to recognize the approach that is most suitable to a particular system and depends on extensive knowledge of that system as well as thorough understanding of the management objectives and continuous stakeholder feedback are critical [62].

Applying a DSS for IWRMP is particularly challenging because water resources systems are generally non-linear and characterized by complex feedback processes [52]. An effective DSS must therefore integrate multiple system processes into a single framework [63, 64].

A quantitative temporal and spatial analysis, such as that associated with the “Realize” phase leading to the “Why” phase (i.e., as in Fig 1 these two phases are associated with the 1^st^ DSS mapping water, food and energy to enable a managerial comparison over the domain nodes, pinpointing “to the extent of…in comparison with”), is not addressed by the existing DSS for IWRMP. This main drawback is rectified in the current developed WFE management tool. Furthermore for the subsequent “How” phase (2^nd^ DSS in Fig 1), based on the lumped parameter modeling approach, we suggest a managerial approach to regulate between nodes WM values associated with their WFE Volumes.

### Water-related DMS for a possible new understanding

In policy-making the importance of researching positions and opinions is embedded in the ability to express a general assessment of a subject [65]. Individuals’ standpoints can explain behavior regarding a given issue and to some degree predict the motives for this behavior. An individual’s perceptions describe his or her attitudes toward a particular topic as well as the intensity and importance of the issue to that individual [66]. A natural gap exists between two aspects of WFE expenditure: objective in terms of actual consumption, and subjective indicating the desired optimal consumption, namely the extent to which a person can afford to consume enough of any of the WFE components to provide for his or her minimum needs versus the extent to which s/he feels that s/he ought to consume them. Examples from Israel are found in a survey by Marke, et al. [64], and in the social survey collected annually by the Israel Central Bureau of Statistics (CBS) [16]. In these surveys using a Likert scale [44] respondents were asked to address, on a quantitative scale of ordinal categories, actual versus desired consumption in a way that suits their needs. Clearly, examination of the objective dimension by itself is insufficient for the learning process about real needs of diverse populations and communities which can potentially spoil the information provided to decision-makers. The latest SHARE [25] survey (65,000 respondents across Europe, including more than 2,400 in Israel) also addressed consumption frequency of food products. Hence, such surveys of attitudes yielding the node residents’ HF motive, should be considered for quantifiable measures toward designing tailor-made policy regulations associated with WFE expenditure.

Complex systems typically encompass myriad components, movements and activities. Some are planned and clearly defined; others may be spontaneous, evolving, or sporadic, such as networking, leadership and pattern development or alliance building.

A predominant concept in the field of complex systems is that of the network structure. The system generally exhibits collective outcome different from the superposition of its separate inputs [67]. Possible state variables dealt by a domain water authority are associated with an “umbrella” of water-related criteria (i.e., criteria and state variables such as: economy and water quantity per quality prices; health and intestinal, lung or influenza diseases; urbanization and inhabitance or area allocation; industry and quantity per quality water allocation; aquifer and its water and contaminant levels; agriculture and crops per water quantity; WFE expenditures per node, each characterized by being dependent on information).

Each state variable can be influencing, influenced or both, in its inter-relation with other criteria related state variables. Influencing nodes may be ordered according to their prominence, which enables to obtain a detailed plan of causes (influencing nodes) and their potential effect (influenced nodes). The causality-detection algorithm used to generate such a direction is derived in Carmi et al. [68]. Pairing between water-related nexus systems with two components (e.g., health water; land water; climate water; urban water; ecology water; economy water; industry water; food water; energy water) enables mutual influences between three connected components (e.g., WFE) and is essential for sustainability and per-household fundamental needs across nodes and over time.

We propose investigating the water-related-information dependent components in terms of one holistic quantity (Volume) and apply information divergence between nodes for their ODE balance equation. It can be shown that the rate of the Volume conforms to Boltzmann entropy, which quantifies the relation between information and Volume as the nexus measure. WFE expenditures are assumed central to the sustenance of life, and as WFE resources are finite, their exploitation accounts for sustainability.

Administered water quantity and the quality of stakeholders’ demands needs to accommodate possible action alternatives (Fig 2), addressed by the domain water authority. Such alternatives may potentially be classified by anthropogenic impact, surface water, groundwater and marginal water.

A spatial DSS differs from a DSS in that it is designed with the aim of solving complex semi-structured spatial problems with multiple criteria. The spatial DSS is based on input of spatial and non-spatial data stored in geodatabases and therefore often combines a geographical information system (GIS) with the DSS [69, 70]. The GIS enables the representation of spatial relations and spatial variability.

As described in Fig 2 we note the complexity of the decision framework system. Each of the possible core, water related, action alternatives is associated with a family of sub-sets one of which (Decisions/Users network regulations) is directly related to the decision maker. The latter implements decisions subject to the WFE nexus to various criteria.

### The WFE Nexus model

The welfare-management approach developed in this section examines temporal mean WFE Volume enabling a first-order assessment for assigning corrections to the domain nodal-network.

Mapping WFE measures can pinpoint to the intervention aimed at balancing sustainability in terms of fundamental needs over the investigated domain. Such a map becomes a practical diagnosis directing the decision-maker to target investments needed to establish an efficient/optimal policy driven management. As WFE measures are subject to temporal and to geospatial scales, we therefore normalize the spatiotemporal water, food and energy expenditures so that these can be mutually compared. Moreover, we describe how WFE expenditures can be utilized as a DMS. It is important to note that provided input data is used only to demonstrate the methodology and thus we do not elaborate on statistical fitness of the data sample size, significance, bias and structure of the raw data.

Let ${\left(\right)}_{i}^{K}$ denote a quantity at time t^K^ where K (= 0, 1, 2,…,κ) is counted by κ consecutive increments and ascribed to node *i*; $W_{i}^{K},\;F_{i}^{K},\;E_{i}^{K}$ denotes expenditures (per any monetary unit) of water, food and energy, respectively, and $\bar{W_{i}^{K}},\;\bar{F_{i}^{K}},\;\bar{E_{i}^{K}}$ their normalized [i.e., $\bar{{\left(\right)}_{i}^{K}}\equiv {\left(\right)}_{i}^{K}/{\left(\right)}_{i}^{0}$] values with respect to their values in the same node and at an initial (k = 0) time level common to all domain nodes. Let $U_{i}^{K}=\bar{W_{i}^{K}}\bar{F_{i}^{K}}\bar{E_{i}^{K}}$ denote the normalized WFE Volume associated with the *i* node along the K time levels. As mentioned before, each of the WFE components are information-dependent and their Volume rate ($\frac{d}{dt}U_{i}^{K}$) can be shown to be equivalent to the form of Boltzmann entropy that expresses a unit of information entropy.

Detailed development appears in our previous manuscript [41]. Briefly, disregarding units, the *W*(*I*)*F*(*I*)*E*(*I*) components are assumed information (*I*(*t*)) time dependent and the WFE Volume reads *U* = *W*(*I*)*F*(*I*)*E*(*I*). The rate of the Volume becomes $\frac{dU}{dt}=(WFE)\;\beta \frac{dI}{dt}$ for which β≡(dWdIW+dFdIF+dEdIE) denotes “compressibility” sum of WFE components respective to their unit measure and relative to change in response to information. We thus obtain $\frac{1}{U}\frac{dU}{dt}=\beta \frac{dI}{dt}$ which after integration for constant *β* value becomes ln *U* = *βI* or *U* = *e*^*βI*^.

It may sometimes be the case that in some network configurations the cumulative WFE Volume rate would resemble more of Shannon’s entropy than that of Boltzmann, if only for the varying inputs and weights at different nodes. We leave this generalization as a topic for future research.

Let $N_{i}^{K}(\leq \kappa )$ denote the number of sequential time steps in the possible evolution period at the *i* node. The WFE temporal mean expenditure Volume ${\tilde{U}}_{i}^{N^{K}}$ (following the first moment notion) after $N_{i}^{K}$ time levels at the *i* node reads

$${\tilde{U}}_{i}^{N^{K}}=\sum_{K=1}^{K=N_{i}^{K}}U_{i}^{K}(t^{K}-t^{0})/\sum_{K=1}^{K=N_{i}^{K}}\left(t^{K}-t^{0}\right).$$ (1)

A relative evolution indicator ${\left(I_{i}^{WFE}\right)}_{rel}$, during the $N_{i}^{K}$ time levels per minimum WFE ${\left(U_{I}^{K}\right)}_{Min}$ Volume at each K time level for the entire investigated domain, can be obtained by

$${\left(I_{i}^{WFE}\right)}_{rel}=\sum_{K=1}^{K=N_{i}^{K}}\omega^{K}U_{i}^{K}/{\left(U_{I}^{K}\right)}_{\mathrm{Min}},\;\omega^{K}\equiv (t^{K}-t^{0})/\sum_{K=1}^{K=N_{i}^{K}}\left(t^{K}-t^{0}\right),\;\omega^{0}=1.$$ (2)

**Fig 3:**
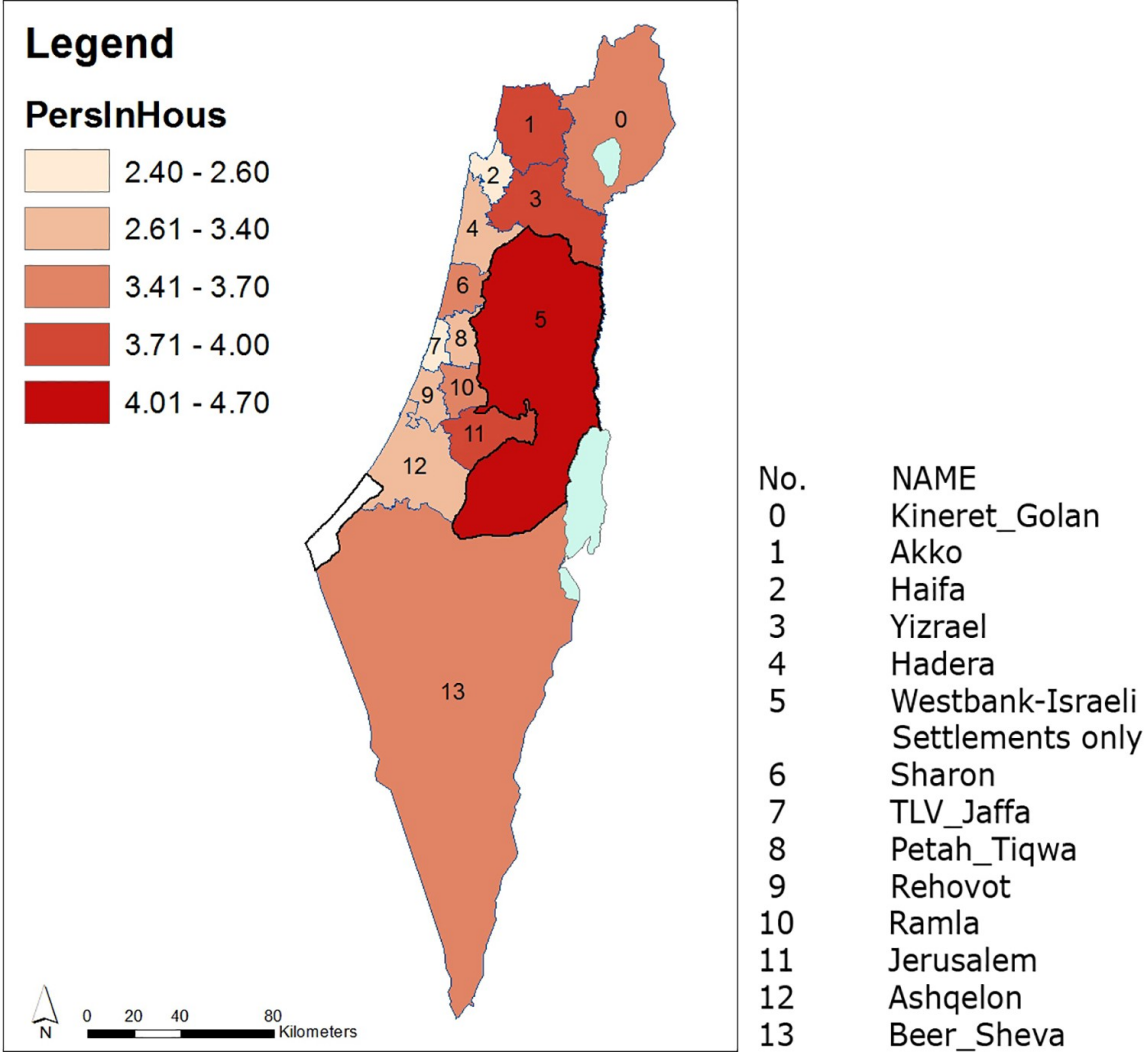
Persons per household for the year 2011, distributed over 14 districts. Districts are numerated in an ascending order from North to South. Values vary slightly for the years 2003 and 2007. All maps in Figs 3–6 have been prepared with ArcGIS Desktop [71]. Country borders and district borders in Figs 3–6 have been downloaded from ArcGIS Online [71]. For country borders see: ESRI, Israel Districts Layer–Ministry of Planning, https://www.arcgis.com/home/item.html?id=2749fad1b0bf45e484d5323e296e37cf (06/03/2021). For country districts see: ESRI, Israel Districts Layer–Ministry of Planning, https://www.arcgis.com/home/item.html?id=927cfe72a31e4a05ab130526c1391acf (06/03/2021).

**Fig 4:**
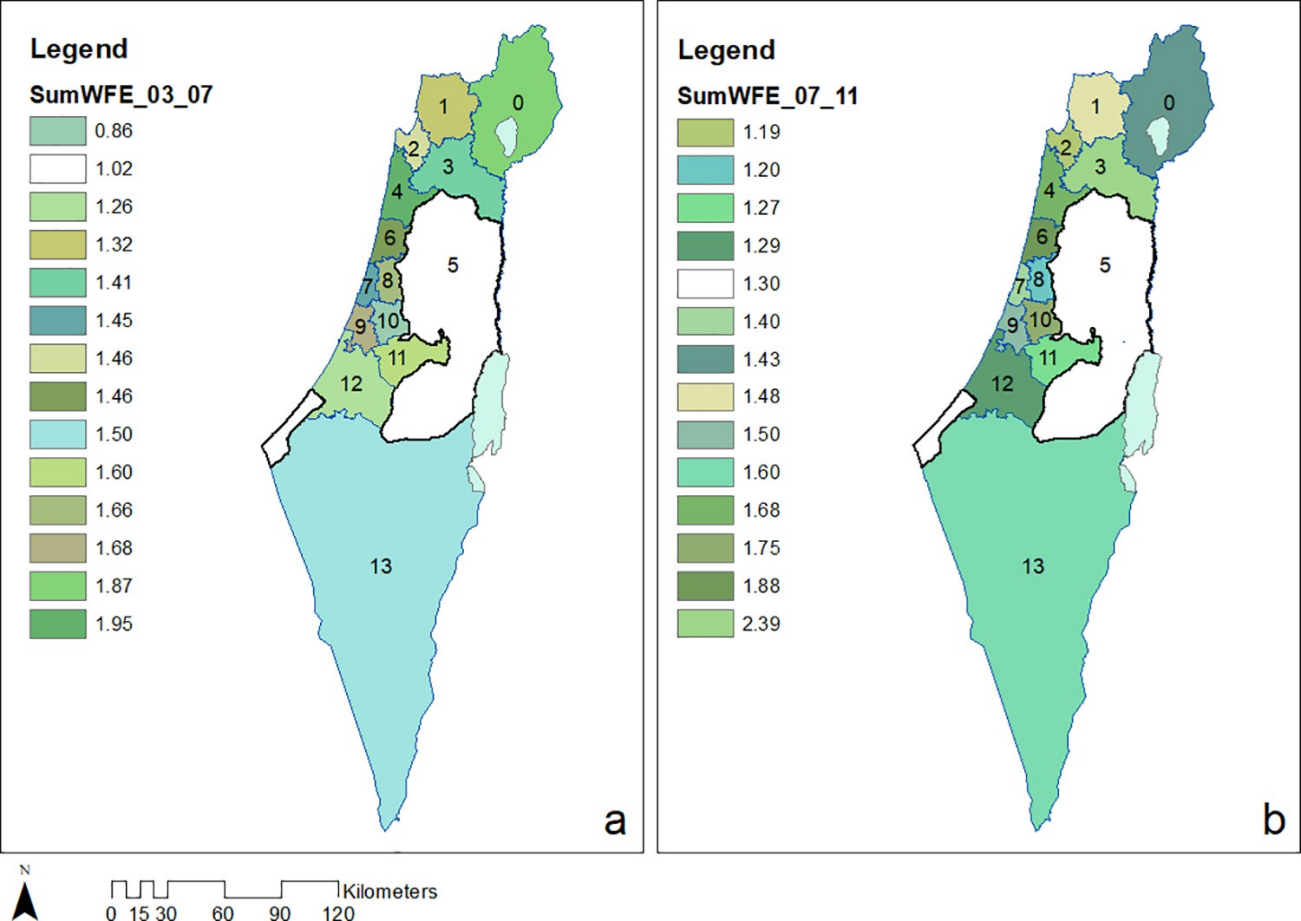
Mean temporal (1), normalized WFE expenditures Volumes per capita over 14 districts for two consecutive periods. (a) 2003–2007 and (b) 2007–2011. Districts enumerations are as in Fig 3. Country borders and district borders have been downloaded from ArcGIS Online [71]. For country borders see: ESRI, Israel Districts Layer–Ministry of Planning, https://www.arcgis.com/home/item.html?id=2749fad1b0bf45e484d5323e296e37cf (06/03/2021). For country districts see: ESRI, Israel Districts Layer–Ministry of Planning, https://www.arcgis.com/home/item.html?id=927cfe72a31e4a05ab130526c1391acf (06/03/2021).

**Fig 5:**
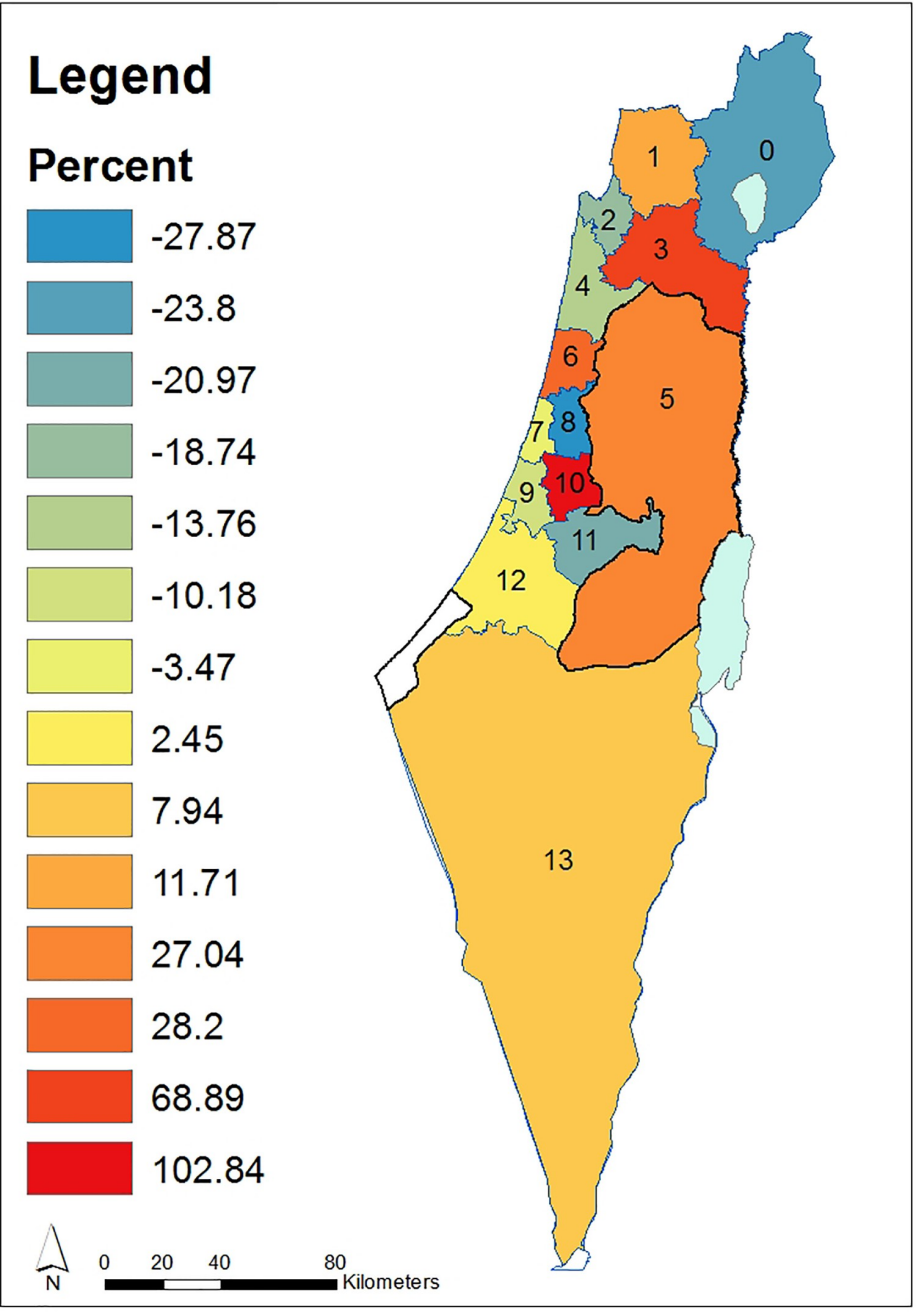
Percentage difference in self-evolution WFE mean Volumes during the period 2007–2011 compared to those of the period 2003–2007. Districts enumerations are as in Fig 3. Country borders and district borders have been downloaded from ArcGIS Online [71]. For country borders see: ESRI, Israel Districts Layer–Ministry of Planning, https://www.arcgis.com/home/item.html?id=2749fad1b0bf45e484d5323e296e37cf (06/03/2021). For country districts see: ESRI, Israel Districts Layer–Ministry of Planning, https://www.arcgis.com/home/item.html?id=927cfe72a31e4a05ab130526c1391acf (06/03/2021).

**Fig 6:**
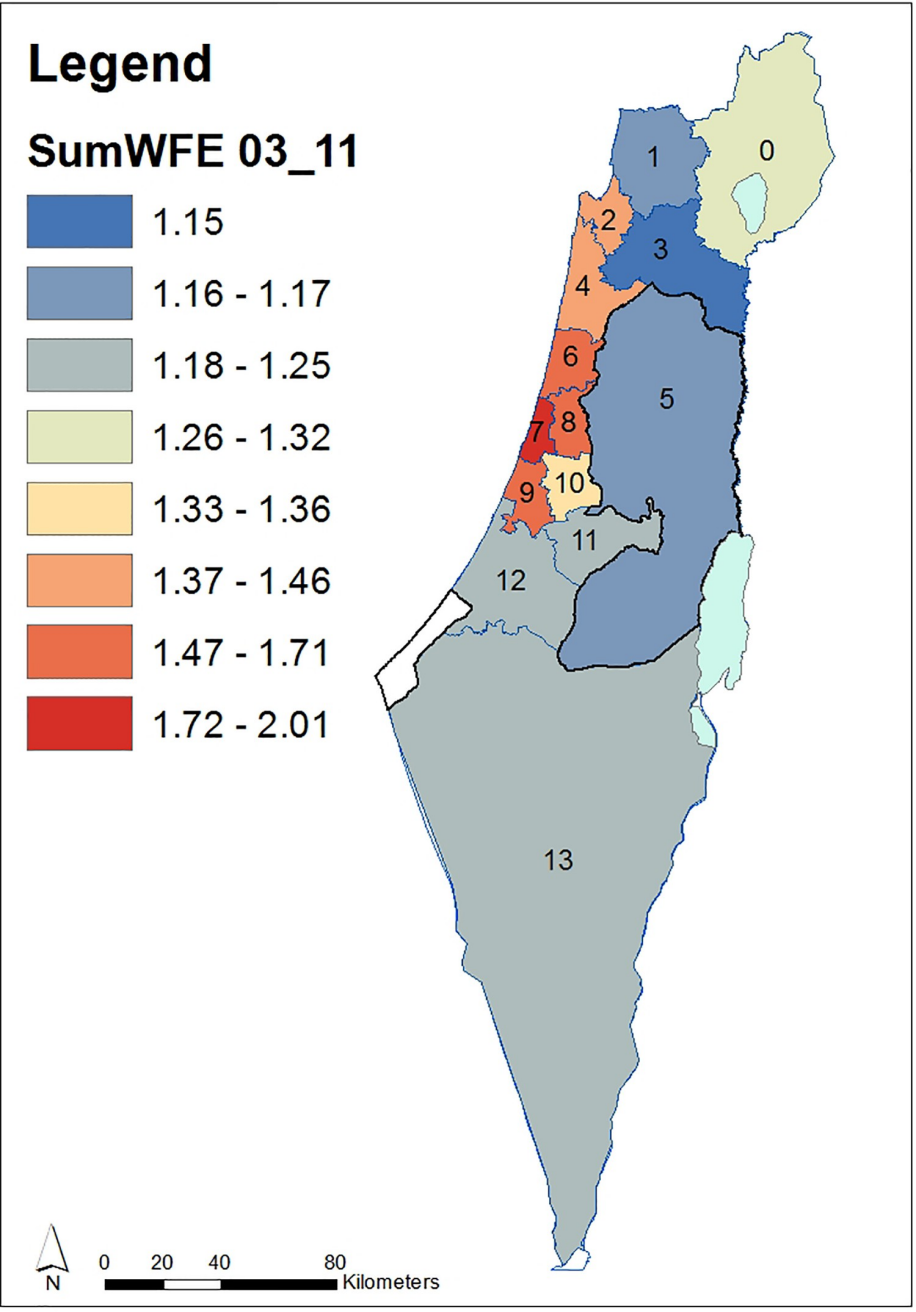
District WFE evolution indicators normalized per year of the trans-country minimal expenditure Volume over the period 2003–2011. Districts enumerations are as in Fig 3. Country borders and district borders have been downloaded from ArcGIS Online [71]. For country borders see: ESRI, Israel Districts Layer–Ministry of Planning, https://www.arcgis.com/home/item.html?id=2749fad1b0bf45e484d5323e296e37cf (06/03/2021). For country districts see: ESRI, Israel Districts Layer–Ministry of Planning, https://www.arcgis.com/home/item.html?id=927cfe72a31e4a05ab130526c1391acf (06/03/2021).

**Fig 7:**
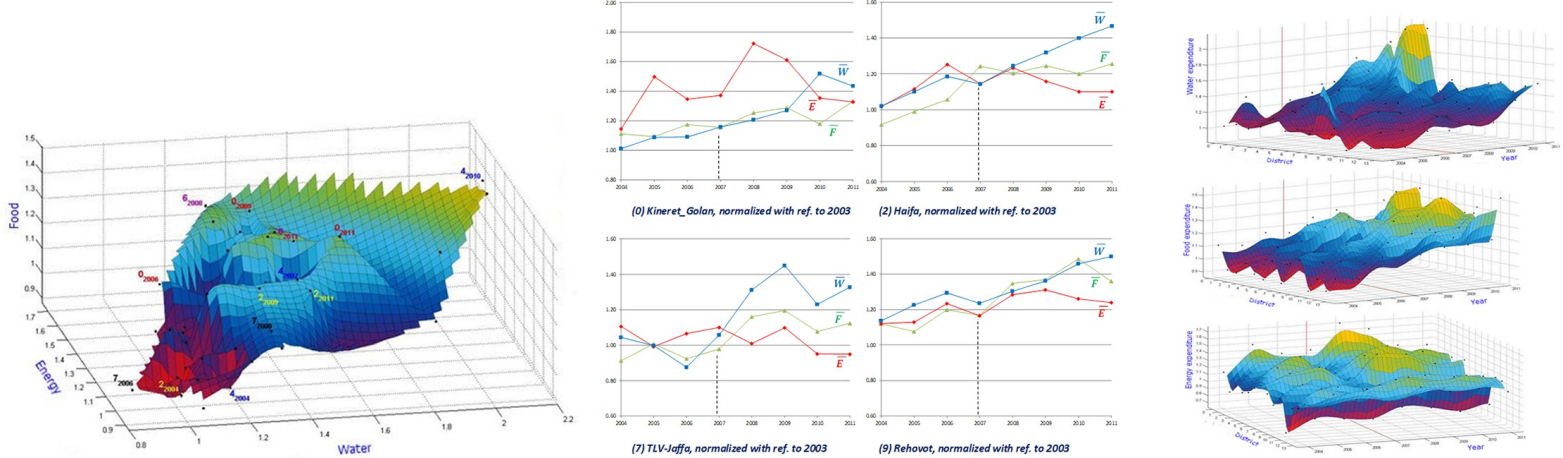
**a.** Interpolated surface of normalized $\bar{W},\;\bar{F}$ and $\bar{E}$ expenditure locus points for Israels’ 14 districts along the continuous years 2004–2011. Note samples of districts’ evolution path. Districts enumerations are as in Fig 3. Surface was computed with Matlab [72]. **b.** Snapshots from the W-F-E surface in Fig 7A, of the normalized components’ evolution trend for four different districts. District labels and their enumerations are as in Fig 3. **c.** Evolution interpolated surfaces of districts’ W, F and E, expenditures, in an enumeration order (Fig 3) from North to South. Note the increase across districts after year 2007. Surfaces were computed with Matlab [72].

We exemplify (1) for a map of mean temporal Volumes (Fig 4) and (2) for the map of evolution indicators demonstrating expenditure for WFE per capita, relative among nodes throughout the entire domain (Fig 6). Data for the 14 administrative nodes (districts), for demonstrating the methodology (S1 Table), was obtained from open-source publications by the Israeli CBS for the years 2003–2011 and is available from the Israeli CBS website at: https://www.cbs.gov.il/en/Pages/default.aspx. It should be noted that data collected for district no. 5 (Figs 3–7) refer only to the Israeli settlements in accordance with the CBS data. Fig 3 describes districts’ distribution of persons per household for years 2003 to 2011. Fig 4 describes maps based on historical data for 14 districts during two consecutive time spans. We note (Fig 4) that the Yizrael district (no. 3) stands out in its self-evolution in the second consecutive time span. As an additional example, when comparing evolution during the 2007–2011 period to that of 2003–2007, we note that mean expenditure Volumes of Petah_Tiqwa (no. 8) and Rehovot (no. 9) have declined, while that of TLV_Jaffa (no. 7) remains unchanged.

The percentage difference in self-evolution between the two periods: years 2003–2007 and years 2007–2011, is described in Fig 5. We note (Fig 5) that among all districts, Ramla (no. 10) distinctively excelled in increasing its WFE Volume.

The map of relative evolution indicators among districts throughout the entire domain, as described by (2), is depicted in Fig 6. We note (Fig 6) that the TLV–Jaffa (no. 7) threshold concerning the WFE indicator is distinctly higher than the other districts throughout years 2003–2011.

In view of the maps depicted in Figs 4–6, the districts are considered as a network of administrative nodes where each *i* node is associated with its WFE Volume at the K time level, and its WM [≡ (*θρU*)_*i*_], in which *θ* denotes the HF motive value and *ρ* denotes the equivalent residents’ density. The HF measure, $\theta_{i}^{K}$, addresses the value for the *i* node at the *K*^*th*^ time level, can be obtained by a survey that addresses residents’ subjective attitude per each expenditure of the WFE components. Let the scale of 1 to 10 express the degree for subjective attitude, ${\left(\overset{˘}{}\right)}_{i}^{K}$, per each of the WFE components, for the *i* node at the *K*^*th*^ time level. Summing over all domain *i* nodes of their normalized [i.e., ${\left(\overset{˘}{}\right)}_{i}^{K}/{\left(\overset{˘}{}\right)}_{i}^{0}$] values with respect to their values in the same node at an initial (*k* = 0) time level common to all domain nodes, reads $\sum_{n=1}^{N}{\left({\overset{˘}{W}}_{n}^{K}/{\overset{˘}{W}}_{n}^{0}\right)}_{i},\;\sum_{n=1}^{N}{\left({\overset{˘}{F}}_{n}^{K}/{\overset{˘}{F}}_{n}^{0}\right)}_{i}$ and $\sum_{n=1}^{N}{\left({\overset{˘}{E}}_{n}^{K}/{\overset{˘}{E}}_{n}^{0}\right)}_{i}$, respectively, for water, food and energy subjective components in which N denotes the number of residents in the *i* node. The mean $\theta_{i}^{K}$ value reads,

$$\theta_{i}^{K}=\frac{1}{N}{\left(\alpha_{W}\sum_{n=1}^{N}{\overset{˘}{W}}_{n}^{K}/{\overset{˘}{W}}_{N}^{0}+\alpha_{F}\sum_{n=1}^{N}{\overset{˘}{F}}_{n}^{K}/{\overset{˘}{F}}_{N}^{0}+\alpha_{E}\sum_{n=1}^{N}{\overset{˘}{E}}_{n}^{K}/{\overset{˘}{E}}_{N}^{0}\right)}_{i};\;{\left(\alpha_{W}+\alpha_{F}+\alpha_{E}\right)}_{i}=1,$$ (3)

in which *α*_*W*_, *α*_*F*_ and *α*_*E*_ are prescribed weights for, respectively, the water food and energy subjective degrees, and their ${\overset{˘}{W}}_{N}^{0}$, ${\overset{˘}{F}}_{N}^{0}$, ${\overset{˘}{E}}_{N}^{0}$ temporal constant values assembled over N residents.

The equivalent residents’ density $\rho_{i}^{K}$ for the *i* node at the *K*^*th*^ time level, accounts for node’s income and for its resident’s areal distribution. Let ${\left(IP\right)}_{i}^{K}[={\left(\sum_{n=1}^{N}{\left(IP\right)}_{n}^{K}\right)}_{i}]$ denote the *i* node’s income (i.e. summed over node’s residents) at the *K*^*th*^ time level. The domain representative income at that time level can be evaluated as a mean value accounting for all domain nodes, reads $\sum_{i}{\left(IP\right)}_{i}^{K}A_{i}/A_{D}$ in which *A*_*i*_ denotes the *i* node area and *A*_*D*_ (= ∑_*i*_*A*_*i*_) denotes the domain area. Its income index $I_{i}^{K}$ at the *K*^*th*^ time level, can be defined as a ratio (i.e. relative node’s income) with reference to the representative domain income, namely $I_{i}^{K}\{\equiv {\left(IP\right)}_{i}^{K}/[\sum_{i}{\left(IP\right)}_{i}^{K}A_{i}/A_{D}]\}$.

The *i* node equivalent resident density *ρ*_*i*_ (i.e., persons per node area or as in Fig 3, persons per household of a node), reads

$$\rho_{i}^{K}\equiv {{\left(I\rho \right)}_{i}^{K}}_{.}$$ (4)

In view of the lumped parameter model we write the system of WM balance equations for all the domain *i* nodes accounting for influxes from *j* nodes and effluxes to *L* nodes (divergence from *i* to *j*). In view of the fact that the WFE Volume rate is equivalent to the form of Boltzmann entropy addressing the transfer of information from a node with high *WM* quantity to one with a low value of such a quantity, we write the ODE balance for WM to read,

$$\frac{d}{dt}{\left(\theta \rho U\right)}_{i}=\sum_{j}R_{ji}[{\left(\theta \rho U\right)}_{j}-{\left(\theta \rho U\right)}_{i}]-\sum_{L}R_{iL}[{\left(\theta \rho U\right)}_{i}-{\left(\theta \rho U\right)}_{L}]+W_{i},$$ (5)

in which *W*_*i*_ denotes a prescribed generation rate (i.e. source) or withdrawal rate (i.e., sink) for the *i* node and *R*_*ji*_ (= *R*_*ij*_) denotes a resistance coefficient between the *i* and *j* nodes. Both *W*_*i*_ and *R*_*ji*_ are calibration factors that are assessed on the basis of historical data. During that past period, one possibility is to consider the *R*_*ji*_ and factors *W*_*i*_ terms as constants to be obtained by an optimization procedure. Another possibility is to address the *R*_*ji*_ as prescribed constants and to obtain the temporal *W*_*i*_ values by solving (5) at time increments throughout the observed period. Following the end of the observed period, the proceeding *W*_*i*_ quantities are superimposed as measures of regulation to achieve a strategic WM balance over all domain nodes, and thus (5) is temporally integrated by an implicit numerical scheme. Moreover, (5) describes a set of first order linear, initial value, ODE’s integrated by, say, an implicit difference scheme. The convergence condition can easily be satisfied for any positive value integration step where the implicit Euler scheme has much better convergence properties when compared with the explicit Euler method. (e.g. see Text_template (nptel.ac.in)).

By virtue of (5), summing over all domain *i* nodes, we have for any time level the condition

$$\sum_{i}\frac{d}{dt}{\left(\theta \rho U\right)}_{i}=\sum_{i}W_{i}$$ (6)

Note that (6) can be interpretated as the balance over one node representing the entire domain. Note also that regulation on the basis of the right hand side of (6) for the K+1 time level, can be assigned a higher or lower value than that of the K time level by respectively increasing (i.e., allocation) or decreasing (i.e., deallocation) any of the *W*_*i*_ quantities or prescribing a combination such that ∑_*i*_*W*_*i*_ = *Const*. with no violation of the total investments over the domain. We maintain that (6), holds no matter how far in space or time, addresses Emmy Noether’s first theorem for which the conservation of the domain WM quantities is due to continuous symmetry, namely invariant under different interventions.

The established management model is addressed by (3) to (6) for predictions subject to management scenarios superimposing source/sink terms to specific nodes. The outcome simulations are beyond the scope of the current manuscript and are reported in Teitelbaum et al. [41], based on historic data and disregarding the HF notion.

Considering further graphical interfaces, addressing historical data as for previous figures, one can draw an expenditure interpolated surface in Fig 7A, for example, an orthogonal coordinate $\bar{W_{i}}\;\mathrm{vs}\;\bar{F_{i}}\;vs\;\bar{E_{i}}$ system associated with the locus points ($\bar{W_{i}^{K}},\;\bar{F_{i}^{K}},\;\bar{E_{i}^{K}}$), of all Israel 14 districts and throughout the increment counts of time levels during the 2004–2011 years, for the analysis of relative inclinations in terms of the normalized WFE components assembeled from time stations.

Fig 7A demonstrates the WFE expenditure surface for the entire investigated domain addressing the 14 districts along the years 2003 to 2011. We note that in Fig 7A, and from some snapshots (Fig 7B), that expenditure for energy is significantly lower than that for water or food.

We note from Fig 7B that the more urbanized districts (most urban to most rural are district nos. 7, 2, 9 and 0) correspond to less expenses for energy in comparison to those for water or food. In all districts, some of which are displayed in Fig 7B, we note a “turning point” around the year 2007. This is in line with the June 2014 issue of “ICTSD Information Note” that refers to a study [73] addressing the severe international food price spike (Figs 3 and 4 in Anania, [73]) that occurred in 2007/2008 and the measures used to accommodate these rising food prices (Fig 1 in Anania, [73], signifying, e.g., the intervention in “price controls/consumer subsidies at the Middle East and North Africa”). Although more research is required to analyze the underlying causes for the "turning point", we maintain that our findings (Fig 7B) fit the Nexus feature of inter dependencies between the components of the WFE expenditures, and could explain the across districts "turning point" around year 2007 as a vivid manifestation of directional information from influencing nodes (U.S. and Thailand) directed to an influenced node (Israel) in a causality directed network (i.e., sharp price increase of wheat and rice in the U.S. and Thailand, respectively). Similarely, as an example, the notion of directed information network of microbe–microbe and host–microbe interactions is suggested in Layeghifard et al. [74]. The interaction between the overall spatiotemporal distributions of the water-food-energy expenditure interpolated surfaces is depicted in Fig 7C, in which the “district” coordinate follows an ascending enumeration (Fig 3) order from North to South. We note (Fig 7C) the effect of the 2007/2008 international food price spike on the water-food-energy expenditure surfaces causing the increase in water, food and energy expenditures onwards from that period (see some districts snapshot evolution trend in Fig 7B). Moreover, we note (Fig 7C) the dominant increase across Israel in the water expenditure surface compared to the food and energy expenditure surfaces. Actually, for each of the water, food and energy surfaces in Fig 7_C_, the notion of information flux across country districts is vivid when addressing variations along a fixed time level. This directed information is the premise of (3) describing the ODE computational tool for balancing the WM (= *θρU*) across country districts, by regulating the source/sink terms in (5).

## Supporting information

S1 Table ILCBS_Household expenditures_2003_2011_per district. All expenditure prices are in Israeli Shekels (ILS).
(XLSX)

## Abbreviations

ABM: Agent-Based Modeling
CBS: Central Bureau of Statistics
DMS: Decision-Making Strategy
DSS: Decision Support System
GIS: Geographical Information Systems
HF: Human Factor
IWRMP: Integrated Water Resource Management and Policy
ODE: Ordinary Differential Equation
SDM: System Dynamics Modeling
WFE: Water-Food-Energy
WM: Welfare mass
